# The Role of Mitochondrial Solute Carriers SLC25 in Cancer Metabolic Reprogramming: Current Insights and Future Perspectives

**DOI:** 10.3390/ijms26010092

**Published:** 2024-12-26

**Authors:** Amer Ahmed, Giorgia Natalia Iaconisi, Daria Di Molfetta, Vincenzo Coppola, Antonello Caponio, Ansu Singh, Aasia Bibi, Loredana Capobianco, Luigi Palmieri, Vincenza Dolce, Giuseppe Fiermonte

**Affiliations:** 1 Department of Biosciences, Biotechnologies and Biopharmaceutics, University of Bari, 70125 Bari, Italy; aa.biotechiub@gmail.com (A.A.); daria.dimolfetta@uniba.it (D.D.M.); antonello.caponio@gmail.com (A.C.); a.singh@studenti.uniba.it (A.S.); luigi.palmieri@uniba.it (L.P.); 2 Department of Biological and Environmental Sciences and Technologies, University of Salento, 73100 Lecce, Italy; giorgianatalia.iaconisi@unisalento.it (G.N.I.); loredana.capobianco@unisalento.it (L.C.); 3 Department of Cancer Biology and Genetics, College of Medicine, The Ohio State University and Arthur G. James Comprehensive Cancer Center, Columbus, OH 43210, USA; vincenzo.coppola@osumc.edu; 4 Department of Translational Biomedicine and Neuroscience, University of Bari, 70125 Bari, Italy; aasiabibi250@gmail.com; 5 Department of Pharmacy, Health and Nutritional Sciences, University of Calabria, 87036 Rende, Italy

**Keywords:** mitochondria, cancer, metabolic reprogramming, mitochondrial carriers, metabolism

## Abstract

Cancer cells undergo remarkable metabolic changes to meet their high energetic and biosynthetic demands. The Warburg effect is the most well-characterized metabolic alteration, driving cancer cells to catabolize glucose through aerobic glycolysis to promote proliferation. Another prominent metabolic hallmark of cancer cells is their increased reliance on glutamine to replenish tricarboxylic acid (TCA) cycle intermediates essential for ATP production, aspartate and fatty acid synthesis, and maintaining redox homeostasis. In this context, mitochondria, which are primarily used to maintain energy homeostasis and support balanced biosynthesis in normal cells, become central organelles for fulfilling the heightened biosynthetic and energetic demands of proliferating cancer cells. Mitochondrial coordination and metabolite exchange with other cellular compartments are crucial. The human SLC25 mitochondrial carrier family, comprising 53 members, plays a pivotal role in transporting TCA intermediates, amino acids, vitamins, nucleotides, and cofactors across the inner mitochondrial membrane, thereby facilitating this cross-talk. Numerous studies have demonstrated that mitochondrial carriers are altered in cancer cells, actively contributing to tumorigenesis. This review comprehensively discusses the role of SLC25 carriers in cancer pathogenesis and metabolic reprogramming based on current experimental evidence. It also highlights the research gaps that need to be addressed in future studies. Understanding the involvement of these carriers in tumorigenesis may provide valuable novel targets for drug development.

## 1. Introduction

Increased cell proliferation expands the energetic and biosynthetic demands of cells. Consequently, cancer cells rewire their metabolism to obtain energy, building blocks for macromolecule synthesis, and reducing equivalents to maintain redox homeostasis [1]. Cancer cells primarily rely on glucose and glutamine as their main carbon sources [2]. Although oxidative phosphorylation of glucose is more energy-efficient, cancer cells often convert glucose to lactate even in the presence of sufficient oxygen, a phenomenon known as aerobic glycolysis or the Warburg effect [3,4,5]. Enhanced glycolysis provides substrates for serine and glycine synthesis, lipid biosynthesis, the hexosamine pathway, and the pentose phosphate pathway (PPP), resulting in increased NADPH production [4]. Lactate is exported via monocarboxylate transporters, where it modulates the tumor microenvironment to promote metastasis, angiogenesis, and immune evasion [6,7]. Another prominent metabolic characteristic of many cancer cells is their strong dependence on glutamine, termed “glutamine addiction” [8]. Cancer cells increase glutaminolysis and utilize glutamine to replenish tricarboxylic acid (TCA) cycle intermediates, essential for energy production, lipid synthesis, and aspartate biosynthesis. Aspartate serves as a precursor for amino acids and nucleotides and supports redox homeostasis [9,10]. The PPP is also upregulated in many tumors to support cell growth and survival by generating ribose-5-phosphate for nucleic acid synthesis and NADPH, which is required for fatty acid synthesis and oxidative stress mitigation [11]. Similarly, one-carbon metabolism is enhanced in cancer cells to support purine and pyrimidine synthesis and methylation reactions [12,13]. Cancer cells also increase fatty acid synthesis and lipogenesis to meet the demand for new membrane formation [14], with enzymes like acetyl-CoA carboxylase, stearoyl-CoA desaturase 1, ATP-citrate lyase, and fatty acid synthase frequently upregulated [15,16]. As a result, metabolic reprogramming is now recognized as a hallmark of cancer [17,18]. It is important to note that metabolic alterations vary among cancer cells, influenced by factors such as driving mutations, nutrient availability, hypoxia, and the tumor microenvironment [19]. Thus, metabolic changes can evolve over time and significantly contribute to tumorigenesis [19,20].

In this complex metabolic landscape, mitochondria play a central role in cancer cell metabolic rewiring, supporting the synthesis of macromolecule precursors (nucleotides, lipids, and amino acids) and oncometabolites [2]. Mitochondrial transporters, located in the inner mitochondrial membrane, facilitate the transport of charged and polar molecules and are key players in these metabolic processes. They enable the selective movement of essential metabolites between mitochondria and the cytosol, facilitating regulated cross-talk between these compartments [21,22]. These transporters move a variety of substrates, including TCA cycle intermediates, amino acids, glutathione, vitamins, cofactors, nucleotides, and minerals [23,24,25,26].

Consequently, they regulate multiple metabolic pathways, such as carbon source oxidation, TCA and urea cycles, the malate–aspartate shuttle, and the synthesis of lipids, pyrimidine nucleotides, and heme [27]. In cancers, the expression of these transporters is altered to promote metabolic adaptations that support cell survival, proliferation, and metastasis [28]. Therefore, targeting mitochondrial carriers is a promising strategy for cancer therapy by disrupting metabolic reprogramming. This review provides a comprehensive analysis of mitochondrial carrier family members’ roles in cancer metabolic rewiring, highlighting gaps in current knowledge and presenting these proteins as potential therapeutic targets for cancer treatment.

## 2. Mitochondrial Carrier System

Human mitochondrial transporters can be classified into two major groups: the canonical mitochondrial solute carrier SLC25 superfamily (MCF) and a non-canonical class, which includes transporters such as the mitochondrial pyruvate carrier (MPC), the serine transporter (SFXN1), and the glutamine carrier SLC1A5_Var. The canonical SLC25 family consists of 53 members, designated as *SLC25A1* through *SLC25A53*, all encoded by nuclear genes. Typically, proteins in this family contain six transmembrane α-helices comprising approximately 300 amino acids, organized into a tripartite structure with three homologous domains (each around 100 amino acids long) that feature a sequence motif PX(D/E)XX(K/R), known as the “carrier signature” [29]. The functions of many of these transporters have been characterized by assessing transport activity in intact mitochondria [21] or through heterologous expression in *E. coli* followed by reconstitution into liposomes [30] as well as via genetic approaches. Notable examples include the recently identified SLC25A39, SLC25A44, SLC25A48, and SLC25A51 [31,32,33,34]. These carriers transport metabolites and cofactors across the inner mitochondrial membrane, including TCA cycle intermediates, amino acids, nucleotides, and cofactors. Most MCF members function as strict exchangers, meaning that the transport of a substrate from one side of the membrane is strictly dependent on the simultaneous transport of another molecule in the opposite direction [35,36,37]. However, a few members, such as those involved in proton-coupled anion symport, operate via a different mechanism [38,39]. Notably, mutations in mitochondrial transporters have been linked to various human metabolic, neurodegenerative, and muscular diseases [40,41,42]. Importantly, several MCF members, such as SLC25A34, SLC25A35, SLC25A45, and SLC25A53, remain functionally orphaned. The following sections present an updated review of the role of SLC25 transporters in cancer-related metabolic reprogramming. A summary of the SLC25 human genes discussed in this review, along with their transport mechanisms and roles in tumor metabolic rewiring, is presented in Supplementary Table S1. Non-canonical transporters are not discussed in detail, except for SLC1A5_Var, due to its significant role in transporting glutamine, a function that complements the transport of glutamine-derived metabolites by some MCF members, which are crucial in cancer metabolism.

## 3. SLC25 Family Members Involved in the Transport of TCA Cycle Intermediates

### 3.1. Citrate Carrier (CIC) or SLC25A1

The mitochondrial citrate carrier (CIC), encoded by the *SLC25A1* gene, is the sole known mitochondrial transporter capable of shuttling citrate, isocitrate, and phosphoenolpyruvate from the mitochondria to the cytosol in exchange for malate [43,44]. CIC can also perform reverse transport, moving citrate from the cytosol into mitochondria [45]. As a key metabolic intermediate of the TCA cycle, citrate is primarily synthesized in mitochondria by citrate synthase from acetyl-CoA and oxaloacetate, with glucose serving as the main source of its carbon skeleton [46]. Citrate plays a vital role in energy production, metabolic regulation, lipid biogenesis, and epigenetic gene regulation through protein acetylation [47,48,49,50]. Thus, CIC serves as a central regulatory player in fatty acid synthesis, glucose metabolism, insulin secretion, and epigenetic modifications [43].

CIC expression is tightly regulated by various transcription factors, including sterol regulatory element-binding protein 1 (SREBP1), Forkhead Box A1 (FOXA1), nuclear factor kappa-light-chain-enhancer of activated B cells (NF-κB), signal transducer and activator of transcription 1 (STAT1), Myc, hypoxia-inducible factor 1α (HIF1α), and peroxisome proliferator-activated receptor gamma (PPAR-γ), which bind to their respective elements in the *SLC25A1* promoter region [43,51,52,53]. Additionally, CIC expression is modulated by the tumor suppressor p53, while wild-type p53 suppresses *SLC25A1* transcription; p53 mutants can upregulate CIC expression at both mRNA and protein levels in H1299 lung cancer cells by recruiting FOXO1 to the *SLC25A1* promoter [51]. Mutations in the *SLC25A1* gene have been associated with metabolic disorders such as type 2 diabetes, obesity, and neurometabolic diseases [54,55]. In cancer, cells upregulate lipogenic enzymes, including citrate synthase and fatty acid synthase, to meet the increased demand for fatty acids and cholesterol synthesis [56]. Cancer cells derive citrate primarily from glutamine through reductive carboxylation or glutaminolysis; although, the extracellular environment also significantly contributes to the citrate pool [57,58]. Consequently, CIC is upregulated in various cancers, including acute myeloid leukemia [59], colorectal [60], breast [51,61], prostate [62], and lung cancers [62,63], and in osteosarcoma [51]. This upregulation is observed at both the mRNA and protein levels and is linked to tumor aggressiveness and poor prognosis [51,59,60,61,64]. Gain-of-function studies have demonstrated that CIC promotes cell proliferation, colony and spheroid formation [63,64], and xenograft tumor growth in mice [51,61,64]. Conversely, CIC knockdown (KO), pharmacological inhibition using benzenetricarboxylate (BTA) or citrate transport protein inhibitors (CTPI-1/2), or the expression of non-functional CIC mutants significantly reduce cell proliferation, colony formation, and tumor spheroid growth [61,63,64]. They also impair cell invasion and migration [63] and enhance sensitivity to ionizing radiation [62] and cisplatin [63], markedly reducing xenograft tumor growth [51,61,64].

**Figure 1:**
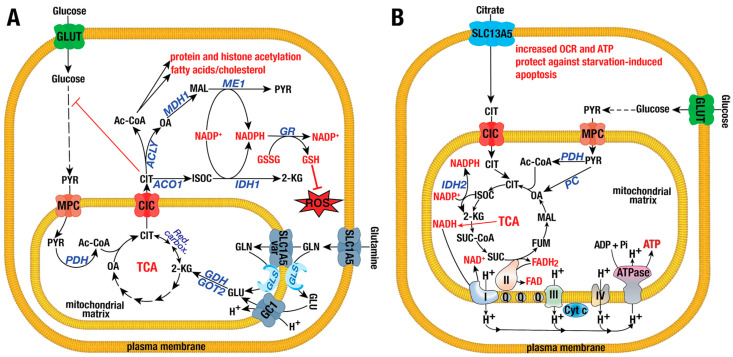
The role of CIC in cancer metabolic reprogramming. (**A**) Under nutrient-sufficient conditions, citrate is synthesized in the mitochondrial matrix from oxaloacetate and acetyl-CoA and is exported to the cytosol by CIC. In the cytosol, citrate is utilized for fatty acid synthesis, protein and histone acetylation, antioxidant regeneration, and ROS elimination. (**B**) During nutrient depletion or high energy demands, citrate is imported from the cytosol into the matrix, where it is catabolized via the tricarboxylic acid cycle to increase cellular respiration, ATP synthesis, and antioxidant production, including NADPH through the IDH2 reaction. ACO1, Aconitase 1; Ac-CoA, Acetyl-CoA; ACLY, ATP-citrate lyase; ATPase, ATP synthase; CIT, Citrate; CIC, Citrate carrier; Cyt C, Cytochrome C; FUM, Fumarate; GC1, Glutamate carrier 1; GDH, Glutamate dehydrogenase; GLN, Glutamine; GLU, Glutamate; GLS, Glutaminase; GLUT, Glucose transporter; GOT2, Matrix glutamate–oxaloacetate transaminase; GR, Glutathione reductase; GSH, Reduced glutathione; GSSG, Oxidized glutathione; IDH1, Isocitrate dehydrogenase 1; IDH2, Isocitrate dehydrogenase 2; ISOC, Isocitrate; MAL, Malate; MDH1, Cytosolic malate dehydrogenase; ME1, Cytosolic malic enzyme; MPC, Mitochondrial pyruvate carrier; OA, Oxaloacetate; OCR, Oxygen consumption rate; PC, Pyruvate carboxylase; PDH, Pyruvate dehydrogenase; PYR, Pyruvate; ROS, Reactive oxygen species; SUC, Succinate; TCA, Tricarboxylic acid cycle; 2-KG, 2-Ketoglutarate.

The protumorigenic role of CIC primarily involves metabolic modulation. Under nutrient-rich conditions, CIC exports citrate from the mitochondrial matrix to the cytosol, promoting fatty acid and lipid biosynthesis without affecting the oxygen consumption rate (OCR) or the activities of respiratory complexes I–V (Figure 1A) [61,64]. In the cytosol, citrate allosterically inhibits phosphofructokinase 1 (PFK1), reducing glycolysis and the Warburg effect while increasing lipogenesis [61,63,65]. Inhibition of CIC by KO or BTA/CTPI-2 decreases cytosolic citrate levels, alleviates PFK1 inhibition, promotes glycolysis, and increases ECAR and lactate production [61,63,65]. In contrast, during metabolic stress (e.g., glucose or glutamine starvation), CIC enhances OCR, ATP production, and the activities of respiratory complexes I–V by reversing the transport of citrate from the cytosol into mitochondria, thereby supporting oxidative phosphorylation (OXPHOS) and protecting against starvation-induced apoptosis (Figure 1B) [62,63,64]. Extracellular citrate uptake is likely the primary source of citrate under these stress conditions [57,58,66]. Cells cultured as 3D spheroids exhibit higher energy demands, with increased OCR and ECAR compared to 2D cultures [67]. In spheroid cells, CIC imports citrate from the cytosol into mitochondria to replenish TCA intermediates like succinate, fumarate, and malate, thereby enhancing OCR [63]. Accordingly, inhibition of CIC using CTPI-2 increases cytosolic citrate levels, which disrupts glycolysis, decreases ECAR and lactate production, and reduces TCA intermediates, ultimately impairing spheroid formation [62,63,64]. Supplementing with citrate or TCA intermediates like succinate and malate can rescue OCR and partially restore spheroid self-renewal [63].

CIC also plays a critical role in maintaining cellular redox balance, particularly under hypoxic conditions, by increasing levels of the antioxidants NADPH and glutathione (Figure 1A). Treatment with BTA or CTPI-2 significantly reduces NADPH and glutathione levels while increasing mitochondrial and cellular ROS [62,63]. Moreover, citrate-derived acetyl-CoA may influence the epigenetic regulation of redox genes, further contributing to CIC’s antioxidant role [68]. Interestingly, CIC’s antioxidant function appears more crucial than its role in fatty acid synthesis. For instance, palmitic acid supplementation does not rescue the cell proliferation defect observed in CIC KO or BTA-treated cells, suggesting that the defect is due to increased ROS levels and loss of mitochondrial membrane potential, which can be rescued by N-acetyl cysteine (NAC) [61]. In summary, CIC is upregulated in various cancers and actively contributes to tumor progression and survival by modulating carbon metabolism based on nutrient availability and energy demands. Under normal nutrient conditions, CIC transports citrate into the cytosol for fatty acid synthesis and antioxidant production. However, under nutrient limitations or high energy demands, CIC shuttles citrate from the cytosol into mitochondria to support energy production through OXPHOS, thereby promoting cell survival.

### 3.2. Dicarboxylate Carrier (DIC) or SLC25A10

The mitochondrial dicarboxylate carrier (DIC), encoded by the *SLC25A10* gene, is responsible for transporting malate or succinate out of the mitochondria in exchange for phosphate, sulfate, or thiosulfate [69]. DIC plays a critical role in sulfur metabolism, fatty acid synthesis [70], glucose-stimulated insulin secretion [71], gluconeogenesis, energy metabolism, and redox homeostasis [72]. It is highly expressed in the liver, kidney, white adipose tissue, and, to a lesser extent, in other tissues [70,73]. In cancer, DIC is upregulated at both the mRNA and protein levels in several solid tumors, including osteosarcoma [74], and lung, breast, ovarian, and gastric cancers [75,76]. Elevated DIC expression is associated with poor prognosis and reduced overall survival [74,75]. Notably, DIC is upregulated in hypoxic tumor regions and is implicated in resistance to radio- and chemotherapy [75]. For instance, DIC expression is increased in anoxia-tolerant NCI-H460 cells compared to normoxic controls [75], which boosts basal and maximal respiration, ATP production, and the NADH/NAD^+^ ratio [75]. Loss-of-function studies reveal that DIC KO significantly reduces cell proliferation and colony formation [74,75,76], enhances the efficacy of ionizing radiation [75], increases sensitivity to cisplatin [76], and induces cell cycle arrest and apoptosis [74]. Moreover, silencing DIC in NCI-H460 cells markedly decreases cellular and mitochondrial levels of GSH while increasing ROS production. Pretreatment with mitoTEMPO, a mitochondrial-targeted antioxidant, or glutathione ethyl ester (GSHEE) rescues DIC KO cells from cell death [75]. In this context, DIC contributes to redox homeostasis, favoring increased antioxidant levels through mechanisms that are not yet fully understood. It is hypothesized that malate transported via DIC is oxidized to pyruvate, reducing NADP^+^ to NADPH through cytosolic malic enzyme activity. Additionally, by transporting the potent oncometabolite succinate, DIC may contribute to tumor growth and progression [77]. Further evidence of DIC’s pro-tumorigenic role has been demonstrated in osteosarcoma, where DIC KO significantly reduces the expression of cyclin E1 (CCNE1) and increases its upstream regulators p21 and p27, suggesting that the DIC–p21/p27–CCNE1 axis plays a role in cell cycle regulation [74,78]. The oncogene CCNE1 is often upregulated or amplified in cancers such as osteosarcoma, lung, and ovarian cancers [79], while the tumor suppressors p21 and p27, frequently lost in various cancers, inhibit CCNE1 to block cell cycle progression to the S-phase [80]. However, the mechanism by which DIC downregulates p21 and p27 while promoting CCNE1 remains unclear. Interestingly, treatment of the lung cancer cell line A549 with metformin, an antidiabetic drug with anticancer properties, results in the downregulation of SLC25A10 at both the mRNA and protein levels [78]. Silencing DIC also reduces A549 cell proliferation, suggesting that DIC may partially mediate the anticancer effects of metformin [78]. Furthermore, the combination of butylmalonate (a DIC inhibitor) with radiotherapy significantly impairs the growth of anoxia-tolerant NCI-H460 xenografts in mice [75]. In summary, DIC is generally upregulated in cancer and is essential for tumor growth and survival. However, the precise mechanisms underlying its oncogenic functions are not yet fully understood but are likely related to its antioxidant role. Further studies are required to determine whether DIC is upregulated in other tumors. More importantly, a deeper understanding of DIC’s role in cancer is needed before considering this carrier as a potential therapeutic target.

### 3.3. 2-Ketoglutarate Carrier (OGC) or SLC25A11

The 2-ketoglutarate carrier (OGC), encoded by the *SLC25A11* gene, is also known as the ketoglutarate/malate antiporter. It exchanges mitochondrial 2-ketoglutarate (2-KG) with cytosolic malate [35]. OGC, along with DIC, has been suggested to transport glutathione [81,82,83,84], though these findings remain controversial [31,85]. The *SLC25A11* gene plays a crucial role in the malate–aspartate shuttle (MAS), the ketoglutarate–citrate (isocitrate) shuttle, glucose oxidation, and energy homeostasis [86]. The MAS involves glutamate-oxaloacetate transaminases (GOT1/2), malate dehydrogenases (MDH1/2), and two mitochondrial carriers: the aspartate–glutamate carrier (AGC) and OGC [87]. The primary role of MAS is to regenerate cytoplasmic NAD^+^ from NADH produced during glycolysis. Mutations in *SLC25A11* have been linked to metastatic paragangliomas [88]. Several lines of evidence support an oncogenic role for OGC in cancer: (i) OGC is upregulated in non-small-cell lung cancer (NSCLC), melanoma [89], hepatocellular carcinoma [90], and in cell line models of these tumors; (ii) loss of function of OGC reduces tumor growth and decreases both the number and overall burden of lung metastases in xenograft mouse models [89,90,91]. Similarly, silencing OGC in tumor-initiating stem cells injected into the liver of NOD/Shi-scid/IL2rγ^−/−^ (NOG) mice significantly decreases tumor growth [90]. In a genetically engineered model, mice heterozygous for *SLC25A11* exhibited a strong reduction in KRAS-mutant-driven lung tumors [89]. (iii) The loss of OGC markedly decreases melanoma cell proliferation, invasion, migration, and colony formation, while increasing cell death in NSCLC [89,91]. Evidence suggests that OGC’s oncogenic effects are mediated by signaling, metabolic, and antioxidant mechanisms. Silencing OGC significantly reduces the expression of mTOR, eukaryotic translation initiation factor (eIF)-4B, and c-Myc proteins, while also abolishing the levels of phosphorylated mTOR and p70SK [89]. The mTOR signaling pathway is critical for cancer cell proliferation and tumor growth [92]. Metabolically, upregulation of OGC is associated with increased OCR, ATP production, and mitochondrial membrane potential (MMP) and coincides with the upregulation of GOT1/2 and MDH1/2, key MAS components [89]. Accordingly, OGC silencing markedly reduces glycolytic flux, TCA cycle intermediates, ATP, mitochondrial NADH, malate levels, MMP, and ECAR, suggesting that cancer cells rely on cytosolic NADH for ATP production [89,90,91]. OGC is upregulated under hypoxia or exposure to the hypoxia mimetic dimethyloxalylglycine (DMOG), whereas it is downregulated upon HIF-1α silencing [90]. The antioxidant effect of OGC has been linked to glutathione (GSH) transport into the matrix [82,93]; although, direct evidence that OGC transports GSH is still lacking [31]. In liver cancer models, OGC KO significantly reduces mitochondrial GSH levels, which can be restored by supplementation with glutathione ethyl ester (GSHEE). Under hypoxic conditions, OGC KO increases ROS levels, oxidative stress-induced cell death, and the amount of active caspase-3, implying apoptosis induction [90]. These effects are reversed by GSHEE supplementation, which also restores OCR and ATP levels, consistent with the protective role of mitochondrial GSH (mtGSH) in maintaining TCA enzyme activity, ATP synthase, and complex IV functions [90,94,95]. In xenograft mouse models, silencing OGC leads to significant depletion of both mtGSH and cytosolic 2-KG [90]. In summary, OGC is upregulated in various cancers, indicating an oncogenic role for this mitochondrial carrier. Its pro-tumorigenic function is likely mediated through increased OCR and ATP production, which supports cell growth and proliferation [96]. However, the significance of mtGSH transport in OGC’s oncogenic role remains controversial [97]. Notably, SLC25A39 has been identified as the primary transporter of GSH into the matrix, and neither DIC nor OGC can rescue mitochondrial GSH content in SLC25A39 KO cells [31]. Although OGC may transport GSH less efficiently than its other substrates, its role in the interplay between GSH and 2-KG may involve uncharacterized epigenetic mechanisms, given that both metabolites are crucial for the regulation of gene expression.

### 3.4. Oxodicarboxylate Carrier (ODC) or SLC25A21

**Figure 2:**
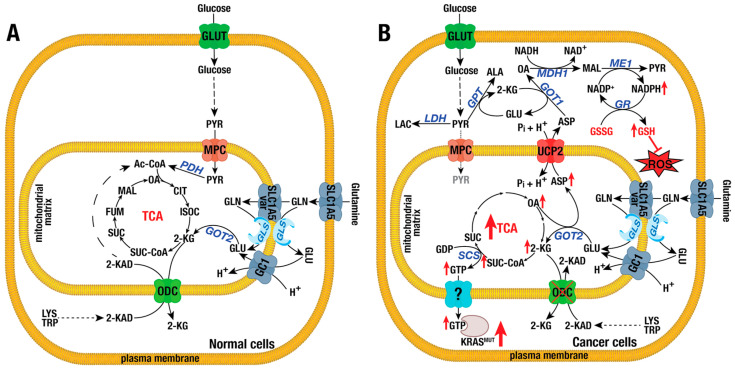
Downregulation of ODC is involved in the metabolic rewiring of glutamine in KRAS-mutant CRC cancer cell lines. (**A**) In normal cells, ODC exchanges cytosolic oxoadipate for mitochondrial 2-KG. (**B**) In cancer cells, downregulation of ODC results in an accumulation of glutamine-derived 2-KG and its downstream metabolites, increased ATP synthesis, and regeneration of NADPH and glutathione by increasing aspartate synthesis and export through UCP2. TCA-derived GTP is increased and likely used in the cytosol for activation of KRAS, which, among other effects, increases glutamine uptake and glutaminolysis. Red arrows indicate increased levels of metabolites, enzyme activities, or activity in metabolic pathways. The question mark indicates that the mitochondrial transporter responsible for the efflux of GTP from the mitochondria remains unidentified. 2-KAD, 2-Ketoadipate; Ac-CoA, Acetyl-CoA; ALA, Alanine; ASP, Aspartate; CIT, Citrate; CRC, Colorectal cancer; Fum, Fumarate; GC1, Glutamate carrier 1; GLU, Glutamate; GLN, Glutamine; GLS, Glutaminase; GLUT, Glucose transporter; GOT1, Cytosolic glutamate–oxaloacetate transaminase; GOT2, Matrix glutamate–oxaloacetate transaminase; GPT, Glutamate–pyruvate transaminase; GR, Glutathione reductase; GSH, Reduced glutathione; GSSG, Oxidized glutathione; ISOC, Isocitrate; LAC, Lactate; LDH, Lactate dehydrogenase; MAL, Malate; MDH1, Cytosolic malate dehydrogenase; ME1, Cytosolic malic enzyme; MPC, Mitochondrial pyruvate carrier; OA, Oxaloacetate; ODC, Oxodicarboxylate carrier; PDH, Pyruvate dehydrogenase complex; PYR, Pyruvate; ROS, Reactive oxygen species; SCS, Succinyl-CoA synthetase; SUC, Succinate; Suc-CoA, Succinyl-CoA; TCA, Tricarboxylic acid cycle; UCP2, Uncoupling protein 2; 2-KG, 2-Ketoglutarate.

ODC, encoded by the *SLC25A21* gene, is highly expressed across various tissues and transports cytosolic 2-ketoadipate (2-KAD) in exchange for mitochondrial 2-KG [98]. In mammals, 2-KAD is produced during the catabolism of lysine, hydroxylysine, and tryptophan. It is then transported into the mitochondrial matrix, where it is further metabolized into acetyl-CoA [98] (Figure 2A). Mutations in ODC result in 2-KAD acidemia and a spinal muscular atrophy-like disease [99]. In cancer, ODC expression is downregulated at both the mRNA and protein levels in bladder cancer [100], colorectal cancer (CRC), pancreatic ductal adenocarcinoma (PDAC) [101], and acute myeloid leukemia [102]. Low ODC expression is associated with poor prognosis and shorter overall survival [100,101,102]. Overexpression of ODC decreases cell proliferation, colony formation, invasion, and migration, while also inducing apoptosis. Conversely, silencing ODC promotes proliferation, colony formation, and cell invasion [100,101]. These tumor-suppressive effects have been recapitulated in xenograft models, where forced expression of ODC reduces tumor growth and metastasis, while its silencing has the opposite effect [100,101]. Interestingly, in CRC cell lines, the tumor-suppressing function of ODC depends on KRAS mutations, as neither loss- nor gain-of-function of ODC affects proliferation or migration in the KRAS wild-type Caco-2 cell line, both in vitro and in vivo [101]. Metabolically, downregulation of ODC contributes to a rewiring of glutamine metabolism, leading to increased glutaminolysis in CRC cells with KRAS mutations. This occurs by preventing the efflux of 2-KG from the mitochondrial matrix into the cytosol (Figure 2B). The use of ^13^C-glutamine as a tracer shows that TCA cycle intermediates derived from glutamine (succinate, fumarate, malate, and oxaloacetate), as well as citrate, are markedly increased, promoting cell proliferation. Furthermore, glutamine deprivation reduces cell proliferation and colony formation, while supplementation with 2-KG restores these phenotypes. Consistently, overexpression of ODC increases mitochondrial 2-KG efflux, thereby reducing cell proliferation and colony formation [100,101]. Downregulation of ODC also affects redox homeostasis by favoring ATP production while reducing the NADP^+^/NADPH ratio and reactive oxygen species (ROS) levels [100,101]. Although reduced ODC expression is not directly caused by KRAS mutations, it increases KRAS-mutant activity. This effect is likely due to increased GTP synthesis via substrate-level phosphorylation in the TCA cycle. In ODC-depleted cells, this leads to enhanced AKT-ERK phosphorylation, which is abrogated by ODC overexpression [101] (Figure 2B). This mechanism is supported by two key lines of evidence. First, inhibiting the conversion of 2-KG into succinate by silencing SUCLG2, a subunit of succinyl-CoA synthetase (SCS), effectively blocks the increase in KRAS activity and significantly reduces colony formation and migration. This outcome mimics the tumor-suppressive effects observed with ODC overexpression in KRAS-mutant CRC cells [101]. Second, attempts to rescue cell proliferation and migration by supplementing with glutamine or 2-KG fail in SUCLG2 KO cells, suggesting that SUCLG2 functions downstream of ODC and is essential for the tumor-suppressive effects of ODC. Furthermore, the downregulation of ODC is linked to reduced activity of 2-KG-dependent DNA demethylases due to the halted efflux of 2-KG [101]. 2-KG activates Ten-Eleven Translocation (TET) DNA demethylases to remove methyl groups from CpG islands within the *SLC25A21* gene promoter, leading to transcriptional activation [101,103]. Accordingly, supplementation with 2-KG in KRAS-mutant CRC cell cultures induces the transcription of ODC [101]. In summary, ODC acts as a tumor suppressor gene that is frequently downregulated in various cancers. It appears to actively participate in regulating glutaminolysis by preventing the efflux of 2-KG from the mitochondrial matrix into the cytosol, thereby enhancing the glutamine flow through the TCA cycle to support ATP production and antioxidant generation (Figure 2B).

## 4. Amino Acid Transporters

### 4.1. Glutamine Carrier or SLC1A5_Var

SLC1A5_Var is a splice variant of the cell membrane sodium-dependent neutral amino acid transporter SLC1A5, which facilitates the transport of glutamine from the cytosol into the mitochondrial matrix [104]. This variant arises due to an alternative transcription site within the first intron of the *SLC1A5* gene, resulting in a transcript that lacks exon 1 and encodes a protein with 339 amino acids [104]. Within mitochondria, glutamine serves as a crucial metabolite for the replenishment of TCA cycle intermediates (anaplerosis) and ATP production. It supports the sustained biosynthesis of nonessential amino acids, proteins, nucleotides, and fatty acids, as well as the generation of the antioxidant NADPH [105]. Cancer cells heavily depend on glutamine to meet their energetic and biosynthetic demands, thereby positioning SLC1A5_Var as a central player in cancer metabolism [8]. Once inside the mitochondria, glutamine is initially converted to glutamate by glutaminase (GLS). Glutamate can then be either deaminated by mitochondrial glutamate dehydrogenase (GDH) or transaminated by glutamic–oxaloacetic transaminase 2 (GOT2) to generate 2-KG, which enters the TCA cycle as a key anaplerotic metabolite [9]. SLC1A5_Var is upregulated in pancreatic, colon, and lung cancer cell lines [104]. Its expression correlates with poor survival rates in various adenocarcinomas [104]. The promoter of *SLC1A5_Var* contains hypoxia response elements, making its expression sensitive to hypoxia-inducible factor 2α (HIF2α). Specifically, HIF2α loss markedly reduces, while its overexpression increases, SLC1A5_Var expression [104]. KO of SLC1A5_Var reduces the proliferation rate, colony formation, and anchorage-independent growth of PDAC cells and induces cell death associated with increased mitochondrial fragmentation and loss of mitochondrial membrane potential [104]. Moreover, SLC1A5_Var mediates hypoxia-induced resistance to gemcitabine, which can be reversed upon SLC1A5_Var KO or GLS inhibition. This resistance is attributed to reduced ROS levels resulting from increased GSH synthesis and NADPH [104,106]. Metabolic tracer analysis reveals that SLC1A5_Var KO decreases levels of glutamine-derived TCA intermediates, such as 2-KG, succinate, fumarate, and malate. It also lowers basal and maximal OCR and ATP production, suggesting a blockade in glutaminolysis [104]. Additionally, SLC1A5_Var KO reduces GSH levels and the NADPH/NADP^+^ ratio, leading to an increase in ROS. Notably, these metabolic alterations are restored upon re-expression of SLC1A5_Var [104].

In contrast, treatment with the GLS inhibitor BPTES or GLS KO nullifies the effects of SLC1A5_Var overexpression, supporting the mitochondrial localization of this enzyme [104]. In xenograft models, SLC1A5_Var KO completely suppresses tumor growth driven by MiaPaCa2 cells [104]. Recent studies by Liu and colleagues have confirmed the presence of this variant at both the mRNA and protein levels in the midbrain of mice and isolated astrocytes [107]. However, there are concerns regarding the methods used for mitochondrial targeting sequence prediction, the antibodies for SLC1A5_Var detection, and the absence of the first 203 amino acids critical for forming the first four transmembrane helices essential for lipid bilayer interactions [108]. These issues raise questions about whether this N-terminal truncated SLC1A5_Var can integrate into membranes and function as a carrier [108]. Moreover, the localization of GLS, which has been debated to reside in the cytosol, further challenges the functional role of SLC1A5_Var in contexts where the glutamine transporter is bypassed, necessitating a glutamate transporter instead [10,22,109,110]. Thus, additional studies, particularly using KO animal models, are needed to confirm the localization of this transporter to the inner mitochondrial membrane and validate its role in glutamine transport before drawing conclusions about its potential contribution to tumorigenesis. Expressing recombinant SLC1A5_Var and reconstituting it into artificial liposomes could help assess its glutamine transport activity and determine its kinetics. In summary, SLC1A5_Var is proposed to transport glutamine into the mitochondrial matrix, where it is utilized to replenish TCA cycle intermediates, support ATP production, and maintain mitochondrial membrane potential, as well as to drive aspartate biosynthesis. Therefore, this carrier plays a significant role in cancer metabolic reprogramming and redox homeostasis. However, its exact localization and overall role in cancer remain to be fully validated.

### 4.2. Glutamate Carrier Isoforms GC1 or SLC25A22 and GC2 or SLC25A18

In humans, glutamate carrier isoforms GC1 and GC2, encoded by the *SLC25A22* and *SLC25A18* genes, respectively, mediate glutamate–proton symport into the mitochondrial matrix [39]. These two carriers differ in their kinetic properties and tissue distribution: (i) GC1 is expressed in the brain, skeletal muscle, liver, and pancreas, while GC2 is mainly found in the brain and testis; (ii) GC1 has a higher Km (~5 mM) for glutamate/H+, similar to the levels measured in intact mitochondria from the liver and kidney, whereas GC2 has a markedly lower Km (~0.26 mM) and shows low expression in many tissues [39]. The glutamate obtained from diet, deamination of glutamine, transamination of 2-KG, and protein degradation is used for synthesizing amino acids like proline, arginine, and ornithine and the antioxidant glutathione. In cancer, glutamate is primarily produced from glutamine by the enzyme GLS. However, depending on the localization of GLS, mitochondrial glutamate carriers may be essential if glutamine is deaminated by cytosolic or intermembrane space-localized GLS or may be negligible if glutamine is transported directly via SLC1A5_Var into the mitochondrial matrix [22,39] (Figure 2). Nevertheless, multiple studies highlight the critical roles played by GCs, especially GC1, in tumorigenesis. GC1 is upregulated at both the mRNA and protein levels in colorectal [109], gallbladder [110], osteosarcoma [111], and pancreatic cancer [109,112]. In these cancers, GC1 is associated with poor prognosis and shorter overall survival [109,111,112]. Overexpression of GC1 increases cell proliferation, colony formation, and anchorage-independent growth in KRAS mutant CRC cell lines [109,110,113]. It also protects PDAC cell lines from ferroptosis [112]. Conversely, silencing GC1 drastically reduces cell proliferation and colony formation [109,111,113], blocks tumor organoid-derived sphere formation [109,110], induces apoptosis and cell cycle arrest, and suppresses cell invasion and migration [109,110,111]. Silencing GC1 also enhances tumor sensitivity to 5-fluorouracil (5-FU) both in vitro and in vivo [109,110] and increases sensitivity to ferroptosis inducers like RSL3 or erastin [112]. Loss of GC1 markedly reduces tumor growth in xenograft models [109,110,111]. Additionally, GC1 KO in intestinal epithelial cells lowers tumor burden in KRAS mutant mice compared to wild-type GC1 mice [113]. GC1 KO reverses KRAS mutant-induced immunosuppression by reducing myeloid-derived suppressor cell (MDSC) recruitment and enhancing CD8+ T-cell infiltration [114]. In CRC, GC1 is critical for KRAS mutant-driven metabolic reprogramming that increases glutamine dependency and the Warburg effect [109]. Silencing GC1 reduces the flow of glutamine into TCA cycle intermediates like succinate, fumarate, malate, and oxaloacetate, while increasing cytosolic 2-KG likely produced by GOT1 [109]. Depletion of glutamine mimics the effects of GC1 KO. Although glutamate supplementation rescues the effects of glutamine depletion, it fails to restore the effects of GC1 KO, indicating that GC1 is essential for transporting glutamine-derived glutamate into the matrix [109,113,115]. The levels of aspartate, asparagine, N-acetyl-aspartate, glycine, and alanine also decrease, suggesting that loss of GC1 impairs both glutaminolysis and aspartate synthesis [109]. Aspartate, which is vital for tumor growth and survival, is elevated in many cancers [109,115]. Metabolic flux analyses reveal that succinate, fumarate, malate, and aspartate peak at 60 min, while asparagine and oxaloacetate reach maximum levels later after incubation with ^13^C-glutamine [109,116]. This suggests that glutamate is first metabolized into aspartate in the mitochondrial matrix, which is then exported to the cytosol and converted into asparagine and oxaloacetate [109,116] (Figure 2B). Supplementing aspartate and succinate can restore proliferation and migration in GC1 KO CRC lines; although, this is blocked by silencing GOT1 [109,115]. Oxaloacetate supplementation can rescue colony formation, while asparagine supplementation restores cell migration [109]. Asparagine also contributes to KRAS mutant-associated immunosuppression in CRC. Mechanistically, GC1 increases asparagine levels, which promotes SRC phosphorylation, leading to ERK/ETS2 signaling and the secretion of CXCL1 to recruit MDSCs via CXCR2 [117]. GC1 KO reduces levels of NAD^+^ and ATP, likely due to reduced oxaloacetate availability for malate synthesis by MDH1 [109]. It also lowers NADPH and glutathione while increasing ROS levels, indicating that GC1 supports tumor growth by maintaining redox balance [109]. In PDAC cells, GC1 inhibits lipid peroxidation and raises NADPH and GSH levels [112]. In this context, GC1 KO caused a reduction in alanine and aspartate levels, together with an increase in glutamate and the NADP^+^/NADPH ratio upon ferroptosis induction, suggesting an impairment of glutamine-derived glutamate metabolism [112]. Supplementation with oxaloacetate, NAC, or GSH restored resistance to ferroptosis-mediated cell death in GC1 KO cells, indicating that GC1 suppresses ferroptosis through changes in NADPH and GSH levels [112]. Forced overexpression of GC1 upregulated G6PD and enhanced NADPH production via PPP. This also increased the levels of stearoyl-CoA 9-desaturase (SCD) and monounsaturated fatty acids (MUFA), both of which reduce the accumulation of oxidized lipids in biomembranes [112]. Furthermore, GC1 KO significantly reduced the levels of ornithine, polyamines, and acetylated polyamines (e.g., putrescine, spermine, N1-acetylputrescine, N1-acetylspermidine, N1-acetylspermine, and N1,N12-diacetylspermine), suggesting that glutamine can contribute to polyamine biosynthesis (known to promote cell growth by acting as oncometabolites) via GC1 [115,118].

The modulation of DNA and histone methylation contributes to GC1’s oncogenic function, likely because DNA demethylases (TET1-3) and histone demethylases (JMJDs) are dioxygenases that require 2-KG as a cofactor and are competitively inhibited by succinate [119]. In KRAS mutant CRC cell lines, levels of 5-hydroxymethylcytosine (a DNA methylation marker) were reduced, implying a decrease in DNA demethylation, which was reversed by the ablation of GC1 or KRAS mutant. Succinate supplementation mimicked the effect of GC1 or KRAS mutant expression [113]. Among the top methylated promoters were protocadherin genes including *PCDHAC2*, *PCDHB7*, *PCDHB15*, *PCDHGA1*, and *PCDHGA6*, which were consequently downregulated [113]. Protocadherin genes (*PCDHαβγ*) act as tumor-suppressing genes by inhibiting Wnt/β-catenin signaling, thus reducing cell growth and viability [113,120]. Consistently, ablation of GC1 decreased the amount of active β-catenin in the nucleus and reduced the levels of β-catenin transcriptional targets, such as LGR5, EPHB2/3, and CD44. Furthermore, histone methylation, such as H3K4 trimethylation at cell stemness-associated genes (*ACSL2*, *CD44*, *EPHB2*, *EPHB3*, and *LGR5*), was increased [113]. In GC1 KO CRC cells, histone demethylases *KDM5* (*KDM5A*, *KDM5B*, and *KDM5C*) demethylated histones around the *LGR5* promoter, leading to its downregulation. Consequently, silencing *KDM5* restored LGR5 expression, which is also a downstream target of the WNT/β-catenin pathway. Silencing both TET1-3 and KDM5C in GC1 KO CRC cells fully restored LGR5 expression [113]. The ablation of the KRAS mutant or GC1 reduced tumor-sphere formation capacity, suggesting a loss of cancer cell stemness [113]. Conversely, succinate supplementation restored LGR5 expression at both the mRNA and protein levels, thereby increasing CRC stemness and tumor-sphere formation. Additionally, GC1 positively correlated with β-catenin and its downstream targets LGR5 and CD44 [113]. In contrast to GC1, GC2 is downregulated in CRC, as observed in The Cancer Genome Atlas (TCGA) and Gene Expression Omnibus (GEO) databases, as well as in other collections of CRC patient samples [121]. Low expression of GC2 is associated with aggressive clinicopathological features and shorter disease-free survival. Metabolically, low expression of GC2 was associated with higher glucose uptake and increased production of ATP and lactate [121]. SLC25A18 expression was inversely correlated with key players of the Warburg effect and Wnt signaling, such as pyruvate kinase M2, lactate dehydrogenase A, Myc, β-catenin, transcription factor 1 and 4, and phosphorylated AKT [121]. The Wnt signaling pathway inhibitor dickkopf inhibitor 1 (DKK1) could reverse phenotypes caused by GC2 downregulation, including increased glucose uptake, lactate production, ATP levels, and reduced cell proliferation [121]. Additionally, GC2 overexpression decreased xenograft tumor growth [121]. In summary, GC1 is upregulated in cancer, particularly in CRC and PDAC. Evidence suggests that GC1 plays a key role in mutant KRAS-driven metabolic reprogramming of cancer cells through multiple mechanisms, including enhanced glutaminolysis, maintenance of redox homeostasis, and epigenetic regulation of genes involved in cell growth, proliferation, and stemness. Further studies are needed to assess GC2 expression in other malignancies and to fully elucidate its role in the metabolic reprogramming of cancer cells.

### 4.3. Aspartate/Glutamate Carrier Isoforms AGC1 or SLC25A12 and AGC2 or SLC25A13

**Figure 3:**
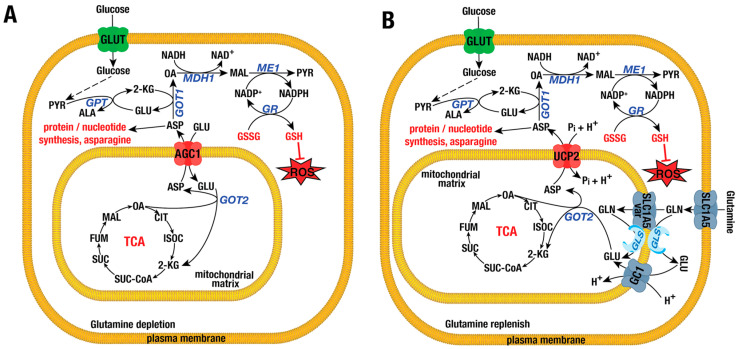
Glutamine level determines the amino acid carrier(s) involved in metabolic rewiring of cancer cells. (**A**) Under glutamine-depleted conditions, UCP2 is downregulated, and the residual aspartate is exported via AGC1 in exchange for cytosolic glutamate due to its low Km value. (**B**) Under glutamine-replete conditions, glutamine enters the mitochondrial matrix through the glutamine carrier SLC1A5_Var or GC1, depending on whether glutaminase is localized in the matrix or the intermembrane space, respectively. Glutamate is then metabolized via the tricarboxylic acid cycle to produce aspartate, which is subsequently transported out to the cytosol by UCP2. In both scenarios, aspartate is utilized by cancer cells to synthesize proteins and nucleic acids or is converted into oxaloacetate by GOT1. Oxaloacetate is reduced to malate by MDH1, and malate is subsequently converted to pyruvate by ME1, generating NADPH for GSSG reduction and ROS control. AGC1, Aspartate/glutamate carrier 1; ALA, Alanine; ASP, Aspartate; CIT, Citrate; FUM, Fumarate; GC1, Glutamate carrier 1; GLU, Glutamate; GLN, Glutamine; GLS, Glutaminase; GLUT, Glucose transporter; GOT1, Cytosolic glutamate–oxaloacetate transaminase; GOT2, Matrix glutamate–oxaloacetate transaminase; GR, Glutathione reductase; GPT, Glutamate–pyruvate transaminase; GSH, Reduced glutathione; GSSG, Oxidized glutathione; ISOC, Isocitrate; MAL, Malate; MDH1, Cytosolic malate dehydrogenase; ME1, Cytosolic malic enzyme; MPC, Mitochondrial pyruvate carrier; OA, Oxaloacetate; PYR, Pyruvate; ROS, Reactive oxygen species; SUC, Succinate; Suc-CoA, Succinyl-CoA; TCA, Tricarboxylic acid cycle; UCP2, Uncoupling protein 2; 2-KG, 2-Ketoglutarate.

The entry of glutamate into mitochondria can also be facilitated by the aspartate/glutamate carriers AGC1 (also known as aralar 1) and AGC2 (also known as citrin), encoded by the *SLC25A12* and *SLC25A13* genes, respectively [122,123]. Both AGC1 and AGC2 function in a Ca^2+^-dependent manner to exchange mitochondrial aspartate for cytosolic glutamate, along with one proton. Ca^2+^ regulates aralar and citrin by binding to a unique long N-terminal extension containing multiple EF-hand motifs exposed to the intermembrane space [124]. These carriers display tissue-specific expression: AGC1 is predominantly found in the heart, brain, and skeletal muscles, while AGC2 is mainly expressed in the liver, gallbladder, and gastrointestinal tract [124]. Together with OGC, AGC1 and AGC2 constitute the MAS, which is responsible for shuttling the glycolytic reducing equivalent NADH from the cytosol into mitochondria [125]. In cancer, AGC1 is upregulated in hepatocellular carcinoma (HCC), PDAC, and lung and ovarian cancers, as well as glioblastoma [126,127]. Loss of AGC1 decreases cell proliferation and cell migration [128,129] and reduces xenograft tumor growth [126,128]. In CRC cell lines, AGC1 KO significantly lowers aspartate and asparagine levels, decreases lactate production, reduces the NAD^+^/NADH ratio, and impairs the OCR [126]. Forced expression of AGC1 or supplementation with pyruvate and aspartate can restore cell proliferation and the NAD^+^/NADH ratio [126,128]. Under conditions of glutamine depletion, AGC1 becomes essential when other transporters, such as UCP2 or GC2, are unable to sufficiently replenish cytosolic aspartate due to their high Km values [22] (Figure 3A,B). Consistently, glutamine depletion or the treatment with the glutaminase inhibitor CB-839 significantly suppresses cell proliferation and increases apoptosis in AGC1 KO CRC cell lines compared to wild-type controls [126]. Supplementation with exogenous aspartate rescues cell growth and viability [126]. In Lewis Lung Cancer (LLC) cells, glutamine depletion upregulates AGC1 expression, suggesting a compensatory mechanism in response to mitochondrial aspartate depletion, which impairs export via other transporters [126]. Both 2-KG and pyruvate can rescue proliferation and survival defects in AGC1 KO cells under glutamine-limited conditions by restoring aspartate levels [126,129]. Collectively, these observations suggest that under glutamine-deprived conditions, aspartate synthesis is reduced, and AGC1 KO cells are unable to export the remaining aspartate due to the high Km of other transporters [126]. Cytosolic aspartate is vital for the biosynthesis of nucleotides, GSH, N-acetyl aspartate, non-essential amino acids (NEAAs) like asparagine, and antioxidants such as NADPH [10,130] (Figure 3). In AGC1 KO LLC cells, glutamine depletion or CB-839 treatment significantly decreases NEAA levels, but supplementation of NEAAs rescues the growth defect. This effect is blocked by the transaminase inhibitor aminooxyacetate (AOA), indicating the importance of transamination. In contrast, aspartate restores cell proliferation and viability in AGC1 KO cells under glutamine-limited conditions, regardless of AOA treatment, suggesting that transamination is not required for aspartate to compensate for glutamine scarcity [126,130]. In summary, AGC1 plays a key role in the MAS to regenerate NAD^+^ and ATP. However, under glutamine depletion, AGC1 is crucial for exporting residual aspartate from mitochondria to the cytosol, where it is used for nucleotide synthesis, NEAA production, and the regeneration of NADPH and GSH (Figure 3A). Further studies are necessary to elucidate the role of AGC1 in cancers such as pancreatic, ovarian, lung, and bone malignancies, particularly regarding its involvement in cancer initiation, progression, and metastasis. The role of AGC2 in cancer remains less explored. Although AGC2 deficiency, leading to type 2 citrullinemia, has been linked to hepatocellular carcinoma [131], it is hypothesized that this might be due to the reactivation of AGC1 [132]. AGC2 is found to be upregulated in several malignancies of the lung, intestine, breast, and skin, correlating with worse overall and disease-free survival [133,134,135]. Overexpression of AGC2 enhances cell proliferation and promotes cell invasion and migration [134]. Conversely, AGC2 loss significantly reduces cell proliferation, cell cycle progression, invasiveness, and mitochondrial count [134]. In line with its MAS function, AGC2 KO in the melanoma cell line MDA-MB-435 and the osteosarcoma cell line U2OS markedly decreases glycolytic rates, lactate levels, cytosolic NAD^+^, mitochondrial NADH, ATP production, and OCR [134]. Additionally, AGC2 KO leads to a substantial reduction in glutamine-derived aspartate and uracil levels [134]. Forced expression of AGC2 boosts glycolytic intermediates, cytosolic NAD^+^, mitochondrial NADH pools, and OCR [134]. In summary, AGC2 is upregulated in cancers and plays an active role in tumorigenesis by supporting efficient energy production and biosynthesis of nucleotides and antioxidants. However, further studies are needed to evaluate its role in other types of cancers.

### 4.4. Uncoupling Protein 2 (UCP2) or SLC25A8

UCP2 is a member of the uncoupling protein sub-family, historically believed to uncouple the electron transport chain (ETC) from ATP synthesis by allowing proton flux from the intermembrane space back into the matrix, thereby dissipating the proton gradient’s energy as heat. It is encoded by the nuclear gene *SLC25A8*, mapped to chromosome 11 [136]. Other members include UCP1 and UCP3-6, encoded by *SLC25A7*, *SLC25A9*, *SLC25A27*, *SLC25A14*, and *SLC25A30* genes, respectively [27,137]. UCP2 is ubiquitously expressed in the gastrointestinal tract, adipose tissue, liver, spleen, pancreas, and immune cells [22,138]. A plethora of transcription factors, microRNAs, and metabolites (e.g., glutamine, GSH) tightly regulate UCP2 levels at the transcriptional, translational, and post-translational levels [139,140,141,142]. UCP2 has a very short half-life of approximately one hour, as it is rapidly targeted for degradation by the ubiquitin–proteasomal system [143]. Several reports suggest an antioxidant role for UCP2, likely by dissipating the proton gradient in the ETC, thus reducing mitochondrial membrane potential and ROS production [144,145]. Our laboratory has demonstrated that recombinant UCP2, reconstituted into artificial liposomes, can transport aspartate, malate, and oxaloacetate to the cytosol in exchange for phosphate plus a proton [146]. In cancer, UCP2 is upregulated in various malignancies, including cancers of the breast [147], colon [148], gallbladder [149], head and neck [150], skin [151], prostate [152], pancreas [153], cervix [154], lung [155], and HCC [156] as well as in leukemia [157]. Higher expression of UCP2 is associated with poor prognosis and shorter overall survival [147,149]. Loss-of-function of UCP2 markedly reduced cell proliferation [147,150,151,153], colony formation [147,150,153], and cell invasion and migration [147,149], while inhibiting tumor organoid or spheroid formation [141,150]. It also led to increased mitochondrial membrane potential, ROS production, and oxidative stress markers [147,156], promoted autophagy [147,156], and induced cell cycle arrest at the G0-G1 phase [149,150]. Additionally, UCP2 KO enhanced the antitumor effects of tamoxifen, cisplatin [147,149], and gemcitabine-induced apoptosis [153,156]. Silencing UCP2 significantly reduced tumor growth in xenograft murine models [141,149,153]. The role of UCP2 in cancer metabolic rewiring has been studied extensively. Our laboratory has shown that UCP2 is involved in KRAS mutant-mediated rewiring of glutamine metabolism in PDAC [153,158]. Specifically, UCP2 KO in KRAS mutant PDAC cell lines led to significant reductions in OCR in the presence of glutamine and abrogated cell proliferation and colony formation [153]. Isotopomer analysis revealed that, under UCP2 KO conditions, glutamine undergoes glutaminolysis, but the TCA intermediates glutamate, aspartate, malate, and 2-KG accumulate in the matrix. This accumulation likely reduces OCR due to inhibitory feedback on ETC complexes, such as the inhibition of complex II by oxaloacetate [153,159]. Aspartate supplementation rescued the cell proliferation defects, whereas glutamate supplementation was effective only under glutamine-depleted conditions [153]. This suggests that under normal glutamine levels, glutamine is transported into the matrix, likely via SLC1A5_Var, or converted into glutamate by glutaminase (GLS), followed by import by GC1 and subsequent catabolism through glutaminolysis. Aspartate is then exported by UCP2 [153] (Figure 2B and Figure 3B). Under glutamine depletion, UCP2’s high Km value prevents effective aspartate transport, which instead occurs via AGC1, bypassing the need for UCP2 [126,153] (Figure 3A). This mechanism may explain why glutamine acts as a potent activator of UCP2 expression, suggesting a feed-forward regulatory role [139]. In T-cell acute lymphoblastic leukemia (T-ALL) cell line HPB-ALL, glutamine upregulates UCP2, and UCP2 silencing leads to TCA intermediate accumulation in the matrix, reducing OCR and cell proliferation rates [157]. UCP2 KO decreases levels of GSH, the GSH/GSSG ratio, and the NADPH/NADP^+^ ratio, while increasing ROS production [153]. Notably, forced expression of mouse UCP2 in UCP2 KO PDAC cells restored cell proliferation, colony formation, ROS levels, and redox balance [153]. The forced expression of UCP2 in murine melanoma B16F10 cells increased OCR while reducing lactate production, ECAR, and pyruvate-derived triglyceride and phospholipid synthesis [160]. Interestingly, UCP2 appears to play divergent roles: as a tumor suppressor in normal cells and as an oncogene in established tumors. In support of this, UCP2 deletion increased intestinal tumor formation in mice. Transcriptomic analysis showed that most genes differentially expressed between UCP2^−/−^ and UCP2^+/+^ mice are involved in primary metabolism and redox homeostasis [148]. In fact, UCP2 KO mice showed higher protein carbonylation (marker of oxidative stress) and GSSG/GSH and lower NADPH levels compared to UCP2 wild-type controls. This suggests that deletion of UCP2 may contribute to tumor initiation by creating a pro-oxidative state [148]. Furthermore, UCP2 loss promoted tumorigenesis by causing the downregulation of G6PD (hence decreasing NADPH) while increasing the rate of phospholipid synthesis by upregulating acetyl-CoA carboxylase and fatty acid synthase [148]. On the other hand, UCP2 was upregulated in established colon cancer induced in an APC^−/+^ mice model and in human colon cancer samples from stage II and stage III cases compared to adjacent normal tissue [148]. Similarly, overexpression of UCP2 in murine epidermal JB6 P+ cells facilitated transformation and increased cell proliferation [161]. In summary, UCP2 plays an active role in glutamine metabolism by transporting aspartate from the matrix to the cytosol for nucleotide synthesis and antioxidant production, thereby promoting tumor cell growth and survival. Consequently, UCP2 exhibits dual functionality in cancer, acting as a tumor suppressor in normal cells but functioning as an oncogene in established tumors, where it may confer resistance to chemotherapy and survival advantages by reducing oxidative stress [149].

### 4.5. Basic Amino Acid Transporter or SLC25A29

SLC25A29, encoded by the nuclear gene *SLC25A29*, was initially identified as a carnitine/acylcarnitine transporter-like protein [162] and an ornithine transporter isoform 3 [163]. However, by reconstituting the human recombinant SLC25A29 protein into artificial liposomes, Porcelli and colleagues found that this protein transports basic amino acids such as arginine, lysine, and, to a lesser extent, histidine and ornithine [30]. Arginine plays a key role in the metabolism of nitric oxide, creatinine, and in the synthesis of polyamines [164]. In cancer, SLC25A29 is upregulated in human cell lines of cervical and prostate cancers, as well as in HCC and PDAC [165,166]. SLC25A29 KO in HeLa cells drastically reduced cell proliferation and migration both in vitro and in xenograft models, whereas its overexpression restored the capacity for cell proliferation and migration [165]. It is thought that SLC25A29 transports arginine into the mitochondrial matrix where it is used by mitochondrial NO synthase to synthesize NO, which in turn inhibits cytochrome oxidase, thus reducing MMP, ROS production, and OCR [165]. This is supported by several lines of evidence: (i) SLC25A29 KO increased OCR, ROS, and MMP, while decreasing glycolysis; (ii) SLC25A29 KO significantly reduced mitochondrial NO levels; (iii) the addition of arginine failed to restore mitochondrial NO levels, while supplementation with the NO donor SNAP did restore them; (iv) culturing HeLa cells in an arginine-depleted medium mimicked the effects of SLC25A29 KO, which was rescued by the addition of arginine or SNAP supplementation [165]. Taken together, this suggests that cancer cells upregulate SLC25A29 expression as a mechanism to sustain redox homeostasis and reprogram metabolism toward aerobic glycolysis, while reducing OXPHOS [165]. Upregulation of SLC25A29 may increase the concentration of arginine in the mitochondrial matrix, which is then utilized by mitochondrial arginase 2 to produce ornithine; the latter serves as a precursor for polyamine synthesis, metabolites known for their tumor-promoting properties. Notably, SLC25A29 is downregulated in endothelial cells within the lung adenocarcinoma tumor microenvironment [167]. This downregulation is associated with faster cell proliferation and migration and reduced apoptosis in endothelial cells [167]. The downregulation of SLC25A29 appears to be mediated by increased histone lactylation of its gene promoter, representing an example of the cross-talk between tumor cells and the tumor microenvironment [167]. In summary, SLC25A29 is dysregulated in cancer, and its expression favors tumor progression and angiogenesis.

### 4.6. Ornithine Carrier Isoforms ORC1 or SLC25A15 and ORC2 or SLC25A2

**Figure 4:**
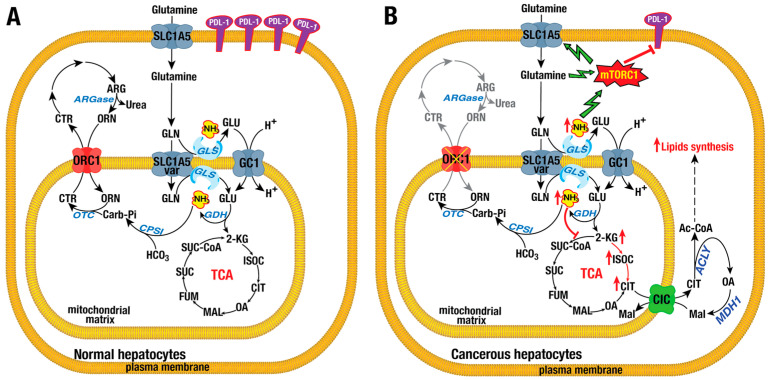
Deficiency of ORC1 actively involved in the metabolic rewiring of glutamine in hepatocellular carcinoma and resistance to Anti-PD-L1 therapy. (**A**) In normal cells, ORC1 exchanges cytosolic ornithine with mitochondrial matrix citrulline as part of the urea cycle. (**B**) In cancer cells, ORC1 is downregulated, leading to an accumulation of ammonia, which in turn downregulates the expression of OGDHL, a subunit of α-ketoglutarate dehydrogenase. This reduces the flow of glutamine through glutaminolysis but increases the reductive carboxylation pathway for citrate synthesis. Citrate exits through CIC to the cytosol, where it is utilized for fatty acid synthesis. Ammonia activates mTORC1, leading to the upregulation of SLC1A5 and increased glutamine uptake, as well as the downregulation of PD-L1, conferring hepatocellular carcinoma with resistance to anti-PD-L1 therapy. Red arrows indicate increased levels of metabolites or activity in metabolic pathways. ACYL, ATP-citrate lyase; ALA, Alanine; ARG, Arginine; Carb-Pi, Carbamoyl phosphate; CIC, Citrate carrier; CIT, Citrate; CPSI, Carbamoyl Phosphate Synthetase 1; CTR, Citrulline; FUM, Fumarate; GC1, Glutamate carrier 1; GDH, Glutamate dehydrogenase; GLU, Glutamate; GLN, Glutamine; GLS, Glutaminase; GSH, Reduced glutathione; GSSG, Oxidized glutathione; HCC, Hepatocellular carcinoma; ISOC, Isocitrate; MAL, Malate; MDH1, Cytosolic malate dehydrogenase; mTORC1, Mechanistic target of rapamycin complex 1; ORC1, Mitochondrial ornithine carrier 1; ORN, Ornithine; OTC, Ornithine carbamoyltranferase; OGDHL, α-Ketoglutarate dehydrogenase subunit; PD-L1, Programmed Cell Death Ligand 1; SUC, Succinate; Suc-CoA, Succinyl-CoA; TCA, Tricarboxylic acid cycle; 2-KG, 2-Ketoglutarate.

The two isoforms of the ornithine carrier, ORC1 and ORC2, encoded by the *SLC25A15* and *SLC25A2* genes, respectively, are known to transport ornithine from the cytosol into the matrix in exchange for citrulline and a proton [168]. ORC1 is expressed in the liver, pancreas, lung, and kidney, while ORC2 expression is restricted to the liver, testis, spleen, lung, and pancreas [169]. Ornithine is a non-protein amino acid primarily produced from arginine through the activities of arginase 1 (ARG1) and arginase 2 (ARG2). The former is localized in the cytosol and mainly expressed in the liver, where it plays a key role in the urea cycle. ARG2, localized in mitochondria, is mainly expressed in the kidney and prostate but is barely detectable in the liver [170]. Although dysregulation of the urea cycle has been widely reported in cancer, studies have mainly focused on the enzymes involved in the urea cycle, with little investigation into the mitochondrial carriers associated with this cycle [171]. Zhang and colleagues showed that ORC1 is downregulated in HCC, which is linked to poor prognosis and shorter overall survival in patients [172]. In HCC cell lines, overexpression of ORC1 reduced cell proliferation, while its silencing increased cell proliferation, invasion, and migration, suggesting a tumor-suppressing function for this carrier [172]. ORC1 KO cells displayed increased intracellular ATP levels without affecting glucose uptake, ECAR, or OCR. An isotopomer analysis of ^13^C-glutamine showed that low expression of ORC1 was associated with increased levels of glutamine, glutamate, and 2-KG [172]. Additionally, palmitic acid and triglyceride levels were higher in ORC1 KO cells, suggesting increased de novo lipogenesis from glutamine via the reductive carboxylation pathway of the TCA cycle [172] (Figure 4A). In ORC1 KO cells, SLC1A5 was upregulated due to enhanced mTORC1 activity, resulting in increased glutamine uptake [172] (Figure 4B). Supporting this, ORC1 KO cells were particularly sensitive to glutamine depletion or treatment with the glutaminase inhibitor BPTES in both 2D and 3D models and exhibited a stronger response to anti-PD-L1 therapy [172]. Additional analysis revealed that ammonia accumulation due to ORC1 downregulation reduced the expression of ketoglutarate dehydrogenase subunit L (OGDHL) [172] (Figure 4B). The metabolic changes induced by ORC1 KO, such as increased ATP, TCA intermediates, and de novo lipogenesis, were reversed by overexpressing OGDHL [172]. In conclusion, ORC1 downregulation in HCC leads to ammonia accumulation, which in turn activates mTORC1, resulting in the upregulation of SLC1A5 and enhanced glutamine uptake (Figure 4B). Additionally, the accumulation of ammonia due to ORC1 downregulation decreases OGDHL levels, directing glutamine metabolism towards the reductive carboxylation pathway and promoting de novo lipogenesis (Figure 4B). Accordingly, OGDHL downregulation fosters HCC by rewiring glutamine metabolism towards lipogenesis and antioxidant synthesis [173]. Further research is needed to explore ORC1 expression in various cancers and to validate its involvement in cancer cell metabolic reprogramming. Additionally, the role of ORC2 remains completely unexplored and warrants investigation.

### 4.7. Mitochondrial Glycine Carrier or SLC25A38

Glycine is imported into the mitochondrial matrix by SLC25A38, a mitochondrial carrier encoded by the nuclear gene *SLC25A38* [174]. In mitochondria, glycine is utilized for the biosynthesis of heme, a cofactor essential for oxygen transport and electron transfer. Heme also functions as a signaling molecule, capable of regulating numerous transcription factors, kinases, ion channels, and micro-RNA processing proteins [175,176]. Furthermore, glycine, along with serine, plays a crucial role in one-carbon metabolism, which provides precursors for the synthesis of macromolecules (nucleotides, proteins, and lipids), antioxidants, and regulates methylation as well as tRNA formylation [177,178]. In cancer, both heme biosynthesis and one-carbon metabolism have multifaceted roles within the tumor microenvironment, promoting tumor growth, immune evasion, angiogenesis, and metastasis [179,180,181]. Therefore, SLC25A38 is expected to play a significant role in cancer progression and metastasis. In fact, SLC25A38 is upregulated in about half of acute lymphoblastic leukemia (ALL) patients and in cell lines such as RPMI 8226, U266, Molt-4, and Jurkat [182]. Consistently, SLC25A38 expression was found to decrease in patients treated with chemotherapy [182]. In contrast, SLC25A38 is downregulated in metastatic uveal melanoma (UM), the most common intraocular tumor in adults [183,184]. This downregulation of SLC25A38 has been identified as an independent predictive and prognostic factor in UM [183]. Correspondingly, loss-of-function of SLC25A38 in UM cell lines, including OCM-1, MUM-2B, and 92-1, promoted cell proliferation and migration, triggered faster tumor growth, and facilitated distant metastasis in xenograft models [183]. The ablation of SLC25A38 causes a reduction in the expression of the E1A-like inhibitor of differentiation 3 (EID3), a potent inhibitor of nuclear receptor-dependent gene transcription that directly binds to and blocks the transcriptional coactivator CREB-binding protein (CBP). This leads to an increased level of free CBP, thereby enhancing HIF1α transcription [183,185]. Consequently, elevated levels of pro-angiogenic cytokines, such as FGF12, TGF8, and TGFα, are expressed and secreted [183]. Overall, the evidence in UM suggests a tumor-suppressing function for SLC25A38, which contrasts with the findings in ALL [183]. These contradictory observations may be due to the role of glycine in maintaining the proliferative status of cancer cells, which appears to be dependent on the specific environment and cell type [186,187].

## 5. Miscellaneous Carriers

### 5.1. NAD^+^ Transporters: SLC25A51 and SLC25A52

SLC25A51 and its isoform SLC25A52, encoded by the homonymous genes, were recently identified as mitochondrial NAD^+^ transporters [34,188,189]. NAD^+^ is a coenzyme that plays a key role in cellular bioenergetics, DNA repair, and redox homeostasis, and acts as a cofactor for sirtuins (NAD^+^-dependent histone deacetylases) [190]. It is synthesized de novo from tryptophan and dietary nicotinic acid and is also recycled from nicotinamide (NAM) and nicotinamide mononucleotide (NMN) via the salvage pathway [191]. In mitochondria, NAD^+^ functions as an electron acceptor for NADH generated by glycolysis or the TCA cycle, thus transferring them to the respiratory chain [192]. Therefore, SLC25A51 is predicted to play a crucial role in cancer pathogenesis by orchestrating cellular metabolism, DNA repair, and epigenetic modifications. Notably, SLC25A51 was found to be upregulated in HCC [193] and colorectal adenocarcinoma at both the mRNA and protein levels [194]. SLC25A51 expression was positively associated with tumor size, vascular invasion, and shorter overall and disease-free survival [193,194]. Compared to wild-type controls, silencing SLC25A51 in HCC cells led to decreased cell proliferation and colony formation, abrogated cell invasion and migration, and significantly reduced tumor growth in xenograft models [193,194]. Conversely, forced overexpression of SLC25A51 promoted proliferation and triggered an invasive and migrative phenotype [193,194]. Additionally, SLC25A51 silencing significantly reduced mitochondrial NAD^+^ levels and increased protein acetylation [193], while inducing a redistribution of the NAD^+^ pool into the nucleus and cytoplasm [195]. In HCC, SLC25A51 transports NAD^+^ to activate mitochondrial Sirtuin 5 (SIRT5), whereas SLC25A51 silencing had the opposite effect [193]. SIRT5 has been reported to regulate glycolysis, the TCA cycle, and the electron transport chain [196,197]. The combined silencing of SLC25A51 and SIRT5 reduced glucose uptake and lactate production, while increasing oxygen consumption rates (OCRs) and the activities of complexes I and II. This suggests that SLC25A51 plays a key role in HCC metabolic rewiring, likely through SIRT5, leading to increased glucose consumption via the glycolysis–Warburg effect and reduced OXPHOS [193]. Consistently, the suppression of glycolysis by replacing glucose with galactose reduced cell proliferation, invasion, and metastasis in cells overexpressing SLC25A51 [193]. In CRC, Li and colleagues showed that SLC25A51 contributed to NAD^+^-dependent SIRT3-mediated deacetylation: SIRT3 overexpression notably decreased acetylation of mitochondrial proteins, particularly superoxide dismutase 2 (SOD2) in SLC25A51 wild-type cells. In contrast, SLC25A51 KO cells showed minimal impact on mitochondrial acetylation levels, indicating that SLC25A51 transports NAD^+^ to the matrix, where it acts as a cofactor for SIRT3 to deacetylate mitochondrial proteins [194]. Among the proteins hyperacetylated upon SLC25A51 KO were pyrroline-5-carboxylate reductase 1 (PYCR1) and Delta-1-pyrroline-5-carboxylate synthase (P5CS), which are tandem enzymes involved in proline synthesis from glutamate, leading to P5CS inhibition and proline depletion [198]. Proline supplementation rescued the proliferation and colony formation defects in SLC25A51-deficient cells [194]. Furthermore, SLC25A51 deficiency markedly decreased the phosphorylation levels of AKT, mTOR, and S6K, which was counteracted by proline replenishment [194,199]. Fludarabine phosphate, a purine anti-metabolite, exhibited potent inhibitory activity against SLC25A51, mimicking the effects of SLC25A51 KO by reducing mitochondrial NAD^+^ and proline content while increasing protein acetylation [194]. Aspirin also potentiated protein hyperacetylation mediated by SLC25A51 KO or fludarabine treatment, significantly reducing tumor cell proliferation and colony formation, as well as inhibiting xenograft growth synergistically with fludarabine [194]. In summary, SLC25A51 is upregulated in cancer and mediates an increase in mitochondrial NAD⁺, which in turn contributes to cancer metabolic reprogramming, mainly through modulating sirtuin proteins. Thus, SLC25A51 plays a crucial role in cancer metabolism, warranting consideration as a potential drug target.

### 5.2. Carnitine/Acylcarnitine Carrier (CAC) or SLC25A20

Mammals use the CAC, encoded by the *SLC25A20* gene, to exchange cytosolic acyl-carnitines for matrix carnitine. Once inside the matrix, acyl-carnitines are converted to acyl-CoAs, which are then oxidized to generate ATP [200,201]. CAC is markedly downregulated in HCC compared to adjacent non-tumor tissues at both the mRNA and protein levels [202]. Conversely, CAC KO significantly increased the proliferation, invasion, and migration of HCC cell lines SNU-368 and SNU-739 [202]. In line with this, miR-132-3p, which targets CAC for downregulation, is upregulated in HCC patient tumors. Moreover, overexpression of miR-132-3p led to CAC downregulation in both SNU-368 and SNU-739 cell lines [202]. Low CAC expression was associated with poor prognosis and shorter overall survival [202]. Notably, CAC expression appears to be independent of hypoxia [202]. Forced overexpression of CAC suppressed cell proliferation and colony formation, induced cell cycle arrest at the G1-to-S phase, and promoted apoptosis. It also decreased cell invasion and migration by suppressing the expression of epithelial–mesenchymal transition (EMT) markers [202]. CAC overexpression further reduced tumor growth and metastasis in xenograft models. At the metabolic level, CAC overexpression increased fatty acid oxidation (FAO), whereas its knockdown reduced FAO and promoted cell proliferation and migration. This suggests that the tumor-suppressive function of CAC is mediated through the enhancement of FAO [202]. In fact, inhibition of FAO by etomoxir reversed the suppression of HCC proliferation and migration mediated by CAC overexpression [202]. Notably, increased FAO has been reported in various types of cancer, where it supports tumor survival, growth, and metastasis [203,204].

### 5.3. S-Adenosylmethionine Carrier (SAMC) or SLC25A26

SAMC, encoded by the nuclear gene *SLC25A26*, catalyzes the exchange of cytosolic S-adenosylmethionine (SAM) for mitochondrial S-adenosylhomocysteine (SAH) [205]. SAM is one of the most important methyl donors needed for the methylation of macromolecules (DNA, RNA, proteins, and lipids) and for polyamine synthesis [206]. Hence, SAM plays a key role in epigenetic modifications through DNA and histone methylation [207]. In mitochondria, SAM is essential for the methylation of mitochondrial targets, including mtDNA and proteins. SAMC mutations have been linked to intramitochondrial methylation deficiencies, which cause respiratory chain alterations, resulting in heart and skeletal muscle myopathies and even neonatal mortality In cancer, SAMC is believed to have a negative impact on tumorigenesis by modulating DNA and protein methylation [208]. Indeed, several studies have shown that SAMC is downregulated in HCC [209] and cervical cancer due to *SLC25A26* gene promoter hypermethylation or the loss of the 3p12-p14 region [210,211,212]. The downregulation of SAMC decreases mitochondrial SAM content and the methylation of mitochondrial DNA, resulting in increased expression of respiratory complex subunits, enhanced oxidative phosphorylation, and elevated ATP production [210]. Furthermore, reduced SAM transport into mitochondria increases the cytosolic content of cysteine and glutathione, markedly lowers levels of ROS, and promotes tumor cell proliferation and survival while also conferring resistance to cisplatin [210].

### 5.4. FAD Carrier: SLC25A32

The FAD carrier, encoded by the nuclear gene *SLC25A32*, is ubiquitously expressed and is believed to transport FAD into the mitochondrial matrix, as it complements the FLX1-mutated yeast strain that displays a mitochondrial FAD transport defect [213,214]. However, other studies have suggested that it may also function in folate transport [215,216,217]. Mitochondrial FAD is crucial for folate metabolism by serving as a cofactor for flavoproteins involved in fatty acid β-oxidation, amino acid metabolism, the respiratory chain, and the glycine cleavage system. Due to the importance of FAD, folate, and one-carbon metabolism, SLC25A32 is expected to play a central role in cancer pathogenesis. This transporter has been found to be upregulated in CRC [218], glioblastoma [219], breast, prostate, ovarian, and liver cancer [220]. The overexpression of SLC25A32 is associated with larger tumor sizes, reduced overall survival, and poor prognosis [218,219,220]. In glioblastoma, SLC25A32 promotes cell proliferation, colony and spheroid formation, and induces an invasive phenotype in the glioblastoma cell line GBM#BG5, primarily through the activation of the PI3K-AKT-mTOR signaling pathway [219]. Silencing SLC25A32 in the pancreatic cell line MiaPaCa-2 significantly decreased proliferation without affecting the folate metabolism cycle [220]. Additionally, SLC25A32 KO increased succinate levels, suggesting a complex II defect, and reduced both basal and maximal respiration, which could be rescued by riboflavin supplementation [220]. Similarly, SLC25A32 KO led to higher production of hydrogen peroxide and a reduced GSH/GSSG ratio [220]. In summary, SLC25A32 is upregulated in various types of cancer and is involved in promoting cell metabolism by increasing the availability of FAD in the mitochondrial matrix while decreasing ROS production. However, further research is necessary to fully elucidate its role in the reprogramming of cancer metabolism.

### 5.5. Iron Carriers: SLC25A28 and SLC25A37

Iron is imported into mitochondria via two transporters, mitoferrin 1 and mitoferrin 2, encoded by the nuclear genes *SLC25A37* and *SLC25A28*, respectively [221,222]. Mitochondria consume a significant portion of cellular iron to synthesize heme and iron–sulfur proteins. Abnormal iron handling in mitochondria has been implicated in obstructive pulmonary diseases and cancer [223]. Mutations in *SLC25A37* and *SLC25A28* are associated with anemia [224] and erythropoietic protoporphyria [225]. In osteosarcoma, both SLC25A37 and SLC25A28 are upregulated, leading to increased mitochondrial iron accumulation, which enhances cell viability, proliferation, migration, and tumor growth in xenograft models. Conversely, silencing SLC25A37 and SLC25A28 reduces mitochondrial iron levels and ROS production [226,227]. The upregulation of these genes is associated with increased ECAR, lactate, and ROS levels, alongside decreased OCR and elevated expression of glycolytic enzymes such as GLUT1, hexokinase 2 (HK2), ALDOA, and LDHA [226]. In the mutant Kras-driven PDAC mouse model (known as KP mice), the ablation of mitophagy regulators PINK1 and PARK2 accelerates tumor growth compared to wild-type controls. In both PINK1 and PARK2 mutant models, lactate levels and ECAR are elevated, while OCR is reduced, suggesting a metabolic shift toward aerobic glycolysis [227]. Consistently, the expression of Glut1, Hk2, Aldoa, Ldha, and HIF1α is upregulated [227]. On the other hand, KO of SLC25A37/28 downregulates these genes and offers protection against pancreatic cancer [227]. In PDAC patients, lower levels of PARK2 and higher levels of SLC25A37 are linked to poor prognosis [227]. PINK1 and PARK2 tightly regulate SLC25A37 and SLC25A28 by targeting them for degradation. The loss of PINK1 and PARK2 leads to the upregulation of these transporters, increasing mitochondrial iron levels and promoting metabolic rewiring toward aerobic glycolysis. However, the role of SLC25A37 and SLC25A28 in cancer remains incompletely understood, warranting further investigation.

## 6. Other Carriers

Several mitochondrial carriers, such as the sulfur oxyanion transporters SLC25A14/UCP5 and SLC25A30/UCP6, the coenzyme A carrier SLC25A42, the glutathione transporter SLC25A39, the branched-chain amino acid transporter SLC25A44, and the phosphate carrier SLC25A3, have not been extensively investigated in the context of cancer. Other carriers with known substrate specificities have only been superficially studied for their potential roles in cancer. For instance, the mitochondrial thiamine pyrophosphate carrier SLC25A19 has been found to be upregulated in HCC and breast cancer cells [228,229]. Upregulation of SLC25A19 is associated with poor prognosis, increased infiltration of immune cells such as macrophages, Th2, and T helper cells, and protection against ferroptosis [228]. In HCC cell lines, SLC25A19 KO significantly reduces cell proliferation and migration while inducing ferroptosis [228]. The pyrimidine nucleotide transporter SLC25A36 is amplified in cervical cancer due to a chromosome 3q gain [230]. In addition, a few carriers, still lacking a known specific transport function, have been linked to various malignancies. SLC25A43, encoded on chromosome Xq24, has been found deleted in HER2-positive breast and lung cancers [231,232,233]. SLC25A43 KO in HER2-positive breast cancer cells (BT-474) increases proliferation and cell cycle progression via enhanced G1-to-S transition, suggesting a role for SLC25A43 as a regulator of the cell cycle through a putative mitochondrial checkpoint [232]. The ablation of SLC25A47 promotes HCC tumorigenesis by activating the mTOR signaling pathway [234]. Furthermore, the mitochondrial carrier homolog 1 (MTCH1), encoded by the *SLC25A49* gene, is highly upregulated in HCC and is associated with metastasis and poor survival [234,235]. MTCH1 deficiency disrupts OXPHOS and increases mitochondrial ROS, initiating retrograde signaling and ultimately triggering ferroptosis [236]. Similarly, MTCH2, encoded by the *SLC25A50* gene, is upregulated in malignant glioma [237], gastric cancer [238], and breast cancer [239] where it promotes cell proliferation, invasion, migration, and cell cycle progression [238,239].

## 7. Concluding Remarks and Future Perspective

Metabolic reprogramming is a hallmark of cancer that drives uncontrolled growth and proliferation. Mitochondria serve as central hubs in these metabolic changes, not only facilitating nutrient oxidation and energy production but also generating precursors for biomolecule synthesis and transmitting retrograde signals to adjust nuclear transcriptional activities. In this context, mitochondrial carriers, particularly the SLC25 family, play significant roles in cancer pathogenesis and energy metabolism. They are key mediators in the cross-talk between mitochondria, other cellular compartments, and the tumor microenvironment. Importantly, mitochondrial carriers are also implicated in epigenetic modifications, such as DNA and histone methylation, histone acetylation, and lactylation.

Our review highlights the extensive literature on the SLC25 carrier family, underscoring how many of these carriers are dysregulated in various cancers. However, their precise role in cancer progression is still not fully understood, leaving several critical questions for future exploration: (i) *characterizing orphan carriers and unknown substrates*—many mitochondrial carriers, including SLC25A16, A34, A35, A41, A43, A45, A47, A49, and A53, remain functionally orphaned, awaiting substrate identification. Conversely, several substrates such as fumarate, oxaloacetate, itaconate, polyamines, and branched-chain keto acids are believed to be crucial in tumor development, yet their corresponding mitochondrial carriers remain unidentified; (ii) *limitations of current in vitro models*—most studies to date have used 2D cell culture models to investigate mitochondrial carriers in cancer. While these models provide valuable insights into cancer genetics, they do not fully recapitulate the complexity of tumors in vivo. Tumors in patients are three-dimensional, containing diverse cell types with varying metabolic states and microenvironmental conditions, such as fluctuations in oxygen and nutrient supply. Thus, caution is needed when interpreting results from 2D cultures. Future research should leverage more sophisticated models, such as 3D organotypic cultures, validated animal models, and patient-derived tissues. Stem-cell-derived organoids, which closely replicate the genetic, cellular, and metabolic characteristics of human cancers, offer nearly physiological platforms that may outperform animal models for certain studies [240]; (iii) *analyzing dysregulation across cancer types*—it is essential to investigate the dysregulation of mitochondrial carriers at both the mRNA and protein levels under consistent conditions, enabling the development of detailed expression profiles for each cancer type. This approach is critical because the activity of one mitochondrial carrier often depends on others. For instance, an upregulation of the citrate carrier could correlate with increased expression of the mitochondrial pyruvate carrier and glutamine/glutamate carriers, given that both pyruvate and glutamine provide the carbon skeletons necessary for citrate synthesis; (iv) *understanding the role of carriers in tumorigenesis*—a key question remains whether the altered expression of mitochondrial carriers is a secondary adaptation to metabolic stress or a driving force in the initial stages of tumor formation. Future studies should elucidate the hierarchy of mitochondrial carrier dysregulation in tumorigenesis, whether it contributes to tumor initiation or serves as an adaptive mechanism for sustained tumor growth and survival; (v) *potential therapeutic strategies*—mitochondrial carriers present promising targets for cancer therapy [241]. However, research on small-molecule inhibitors is still limited, partly due to the complexity of high-throughput inhibition assays and the limited understanding of these carriers’ roles in cancer. For example, the UCP2 inhibitor genipin has shown broad activity against cancer cell lines, but it still requires validation in vivo [242]. Similarly, inhibitors targeting the citrate carrier have demonstrated potential anticancer effects and warrant further investigation for clinical applications [243,244]. However, other carriers remain unexplored in the context of cancer therapy. Future studies should focus on discovering potent compounds targeting carriers directly involved in cancer pathogenesis.

While small-molecule inhibitors face challenges, such as high screening costs, potential off-target effects, and toxicity, gene-based therapies offer an alternative. For instance, small interfering RNA (siRNA) strategies can selectively inhibit target mRNA, minimizing off-target impacts. Notably, in vivo delivery of nanoparticle-associated siRNA targeting SLC25A22, combined with anti-PD1 therapy, has shown efficacy in reducing tumor growth in KRAS mutant colorectal cancer models [114]. In summary, advancing our understanding of mitochondrial carrier functions and their regulatory mechanisms in cancer will be essential for developing novel therapeutic strategies. Continued research could unlock the potential of targeting these carriers, whether through pharmacological inhibitors or gene-based therapies, to improve cancer treatment outcomes.

## Supplementary Materials

The following supporting information can be downloaded at https://www.mdpi.com/article/10.3390/ijms26010092/s1.

## Abbreviations

2-KAD, 2-Ketoadipate; 2-KG, 2-Ketoglutarate; 5-FU, 5-Fluorouracil; AGC, Aspartate/Glutamate Carrier; ALL, Acute Lymphoblastic Leukemia; AOA, Aminooxyacetate; BPTES, Bis-2-(5-phenylacetamido-1,2,4-thiadiazol-2-yl)ethyl sulfide; CAC, Carnitine/Acylcarnitine Carrier; CBP, CREB-Binding Protein; CCNE1, Cyclin E1; CD44, Cluster of Differentiation 44; CIC, Citrate Carrier; CRC, Colorectal Cancer; DIC, Dicarboxylate Carrier; DMOG, Dimethyloxalylglycine; DKK1, Dickkopf-related Protein 1; eIF, Eukaryotic Translation Initiation Factor; EMT, Epithelial–Mesenchymal Transition; ETC, Electron Transport Chain; FAO, Fatty Acid Oxidation; FGF, Fibroblast Growth Factor; FOXA1, Forkhead Box A1; G6PD, Glucose-6-Phosphate Dehydrogenase; GEO, Gene Expression Omnibus; GLS, Glutaminase; GLUT1, Glucose Transporter Type 1; GOT1/2, Glutamate–Oxaloacetate Transaminase 1 and 2; GSH, Reduced Glutathione; GSHEE, Glutathione Ethyl Ester; HCC, Hepatocellular Carcinoma; HIF, Hypoxia-Inducible Factor; Hk2, Hexokinase 2; HMB, High Mitochondrial Binding; HPB-ALL, Human Precursor B Acute Lymphoblastic Leukemia; KDM5, Lysine-Specific Demethylase 5; KO, Knockout; KP, Kras-driven Pancreatic Cancer; LDHA, Lactate Dehydrogenase A; LLC, Lewis Lung Carcinoma; LGR5, Leucine-rich Repeat-Containing G-Protein-Coupled Receptor 5; MAS, Malate–Aspartate Shuttle; MCF, Mitochondrial Carrier Family; MDH1/2, Malate Dehydrogenase 1 and 2; MMP, Mitochondrial Membrane Potential; mTOR, Mechanistic Target of Rapamycin; MUFA, Monounsaturated Fatty Acids; NAM, Nicotinamide; NADPH, Nicotinamide Adenine Dinucleotide Phosphate (reduced form); NF-κB, Nuclear Factor Kappa-Light-Chain-Enhancer of Activated B Cells; NSCLC, Non-Small-Cell Lung Cancer; NOG, NOD/Shi-scid/IL2rγ^-/-^; ODC, Oxodicarboxylate Carrier; OGC, 2-Ketoglutarate Carrier; OXPHOS, Oxidative Phosphorylation; PCDH, Protocadherin; PDAC, Pancreatic Ductal Adenocarcinoma; PFK1, Phosphofructokinase 1; PI3K, Phosphoinositide 3-Kinase; PPP, Pentose Phosphate Pathway; PYCR1, Pyrroline-5-carboxylate Reductase 1; ROS, Reactive Oxygen Species; S6K, Ribosomal Protein S6 Kinase; SCD, Stearoyl-CoA Desaturase; SCS, Succinyl-CoA Synthetase; SFXN1, Serine Transporter; SIRT, Sirtuin; SNAP, S-Nitroso-N-Acetyl-DL-Penicillamine; SRC, Steroid Receptor Coactivator; SREBP1, Sterol Regulatory Element-Binding Protein 1; STAT1, Signal Transducer and Activator of Transcription 1; T-ALL, T-Cell Acute Lymphoblastic Leukemia; TCA, Tricarboxylic Acid (Cycle); TET, Ten-Eleven Translocation (DNA Demethylase); Th2, T-helper Type 2 Cell; UCP2, Uncoupling Protein 2; UM, Uveal Melanoma.
