# Supplementary material for: The Role of Mitochondrial Solute Carriers SLC25 in Cancer Metabolic Reprogramming: Current Insights and Future Perspectives
Source: Int J Mol Sci. 2024 Dec 26;26(1):92. doi: 10.3390/ijms26010092 (PMC11719790; doi:10.3390/ijms26010092)
Supplement: Supplementary file 1 [file ijms-26-00092-s001.zip › Supplementary Table S1.pdf]

Table S1. Mitochondrial carrier family members involved in cancer metabolism

Gene	Chromosomal location	Protein name (Symbol)	Transport type/ coupling ions*	Tumor implications	Refs.
SLC25A1	22q11.21	Citrate carrier (CIC)	E/Citrate plus H^+/malate	CIC is upregulated in CRC, lung cancers etc. It promotes cell proliferation and metastasis and protects against apoptosis under nutrient deficiency. It also exerts a profound antioxidant effect and promotes lipogenesis.	[59-65]
SLC25A8	11q13.4	Uncoupling protein 2 (UCP2)	E/aspartate or malate/phosphate plus H^+	UCP2 is upregulated in several cancers including CRC and PDAC. It is involved in glutamine metabolism by transporting aspartate into the cytosol for nucleotide synthesis and antioxidant production, thereby promoting tumor cell growth and survival.	[147-157]
SLC25A10	17q25.3	Dicarboxylate carrier (DIC)	E/malate/phosphate	DIC is upregulated in osteosarcoma, lung, breast, ovarian, and gastric cancers and associated with poor prognosis and overall survival. It was implicated in resistance to radio- and chemotherapy and reduces ROS level.	[74-76]
SLC25A11	17p13.2	Ketoglutarate /malate carrier (OGC)	E/2-Ketoglutarate/ malate	OGC is upregulated in NSCLC, melanoma, and HCC. It promotes tumor growth and	[89-91]

				metastases in xenograft mouse models. It increases OCR and ATP production, and reduces ROS.	
SLC25A12	2q31.1	Aspartate/glutamate carrier 1 (Aralar, AGC1)	E/aspartate/ glutamate plus H^+	AGC1 is upregulated in HCC, PDAC, lung and ovarian cancers and involved in oxidation of glycolysis-derived NADH. Under glutamine depleted condition, it also exports the residual aspartate from mitochondria to the cytosol for nucleotide synthesis, regeneration of NADPH and GSH.	[126-129]
SLC25A13	7q21.3	Aspartate/ glutamate carrier 2 (Citrin, AGC2)	E/aspartate/ glutamate plus H^+	AGC2 is upregulated in lung, intestine, breast, and skin cancers, and is associated with worse overall survival. It promotes glycolysis and increases mitochondrial NADH, ATP production, and OCR.	[133-135]
SLC25A15	13q14.11	Ornithine/citrulline carrier 1 (ORNT1 or ORC1)	E/ornithine/citrulline plus H^+; E/ornithine/H^+	ORNT1 is downregulated in HCC and is linked to poor prognosis and shorter survival. It contributed to glutamine metabolic rewiring towards fatty acid synthesis.	[172]
SLC25A18	22q11.21	Glutamate carrier 2 (GC2)	C/glutamate plus H^+, E/glutamate/OH^-	GC2 is downregulated in CRC and is associated with tumor aggressiveness and shorter	[121]

				survival. It promotes glucose uptake, lactate production (Warburg effect), and Wnt signalling.	
SLC25A19	17q25.1	Thiamine pyrophosphate carrier (TPC)	E/Thiamine pyrophosphate/ATP	TPC is upregulated in HCC and breast cancer cells and was associated with poor prognosis, increased immune infiltration, and reduced ferroptosis.	[228, 229]
SLC25A20	3p21.31	Carnitine/ acylcarnitine carrier (CAC)	E/carnitine/ acylcarnitine	CAC is downregulated in HCC. Its downregulation promotes cell proliferation and metastasis and inhibits fatty acid oxidation.	[202]
SLC25A21	14q13.3	Oxodicarboxylate carrier (ODC)	E/2-ketoadipate/ 2-ketoglutarate	ODC is downregulated in CRC, PDAC, and bladder cancer, and this is associated with increased cell proliferation and migration, enhanced glutaminolysis, ATP production, an elevated NADPH/NADP ratio, and reduced ROS levels.	[100-102]
SLC25A22	11p15.5	Glutamate carrier 1 (GC1)	C/glutamate plus H^+, E/glutamate/OH^-	GC1 is upregulated in colorectal, gallbladder, osteosarcoma, and pancreatic cancers and was associated with poor prognosis and survival. It plays a key role in metabolic reprogramming through multiple mechanisms: enhanced glutaminolysis,	[109-113]

				maintenance of redox homeostasis, epigenetic regulation of genes expression and synthesis of polyamine	
SLC25A26	3p14.1	S-adenosylmethionine carrier (SAMC)	E/S/adenosylmethionine/ S-adenosylhomocysteine	SAMC is downregulated in HCC and cervical cancer, thus decreasing mitochondrial SAM content and the methylation of mitochondrial DNA. It promotes oxidative phosphorylation, and ATP production, while increases the cytosolic content of cysteine and glutathione.	[209-212]
SLC25A28	10q24.2	Mitoferrin 2 (MFRN2)	(?)/iron	MFRN2 is upregulated in osteosarcoma. It drives cell proliferation, migration, and tumor growth by increasing glycolytic flow, ECAR, lactate levels, while decreasing OCR.	[226]
SLC25A29	14q32.2	Basic amino acid transporter (BAC, CACL)	U/arginine<br>U/lysine<br>U/histidine<br>U/ornithine	BAC is upregulated in HCC, PDAC, cervical and prostate cancers. It increases cell proliferation and migration, reduces MMP, ROS production, and OCR while increases glycolytic flow and Warburg effect.	[165]
SLC25A32	8q22.3	Folate carrier (MFT)	(?)/FAD	MFT is upregulated in CRC, glioblastoma, breast, prostate, ovarian, and liver cancers. It promotes cell proliferation and	[218-220]

				metastasis, enhanced cell respiration by increasing FAD availability and decreasing ROS production.	
SLC25A37	8p21.2	Mitoferrin 1 (MFRN1)	(?)/iron	MFRN1 is upregulated in osteosarcoma and PDAC, promoting tumor growth and metastasis by enhancing the expression of glycolytic enzymes (hexokinase 2, ALDOA, and LDHA) and the glucose transporter GLUT1, thereby driving the Warburg effect.	[226, 277]
SLC25A38	3p22.1	Glycine transporter (GlyC)	§U/glycine	GlyC is downregulated in metastatic uveal melanoma. Its downregulation triggers cell proliferation and tumor growth and increases transcription of HIF1α and hence pro-angiogenic cytokines, like FGF12, TGF8, and TGFα.	[183]
SLC25A43	Xq24	Mitochondrial solute carrier 43	Unknown/Unknown	SLC25A43 is deleted in HER2-positive breast and lung cancers leading to increased proliferation and cell cycle progression.	[231-233]
SLC25A49	6p21.2	Mitochondrial carrier homolog 1 (MTCH1)	Unknown/Unknown	MTCH1 is upregulated in HCC and is associated with metastasis and poor survival. It maintains OXPHOS and ROS	[235, 236]

				levels and protects against ferroptosis.	
SLC25A50	11p11.2	Mitochondrial carrier homolog 2 (MTCH2)	Unknown/Unknown	MTCH2 is upregulated in malignant glioma, gastric, and breast cancers. It promotes cell proliferation, invasion, migration, and cell cycle progression.	[237-239]
SLC25A51	9p13.1	NAD^+ transporter (MCART)	(?)/NAD^+	MCART1 is upregulated in HCC and CRC, and its expression is associated with larger tumor size and shorter overall survival. It enhances mitochondrial NAD+ levels and sirtuin protein activity, leading to increased glucose uptake, promotion of the Warburg effect, and a decrease in OCR.	[193, 194]

E, Exchanger; C, Cotrasporter; U, Uniporter; (?) Unknown transport type; §, verified only with the yeast ortholog.
